# Recent Advances in Poly(vinylidene fluoride) and Its Copolymers for Lithium-Ion Battery Separators

**DOI:** 10.3390/membranes8030045

**Published:** 2018-07-19

**Authors:** João C. Barbosa, José P. Dias, Senentxu Lanceros-Méndez, Carlos M. Costa

**Affiliations:** 1 Centro de Física, Universidade do Minho, 4710-057 Braga, Portugal; joaocpbarbosa@live.com.pt (J.C.B.); jmpedrodias@gmail.com (J.P.D.); cmscosta@fisica.uminho.pt (C.M.C.); 2 BCMaterials, Basque Center Centre for Materials, Applications and Nanostructures, UPV/EHU Science Park, 48940 Leioa, Spain; 3 IKERBASQUE, Basque Foundation for Science, 48013 Bilbao, Spain; 4 Centro de Química, Universidade do Minho, 4710-057 Braga, Portugal

**Keywords:** PVDF, copolymers, battery separator, lithium-ion batteries

## Abstract

The separator membrane is an essential component of lithium-ion batteries, separating the anode and cathode, and controlling the number and mobility of the lithium ions. Among the polymer matrices most commonly investigated for battery separators are poly(vinylidene fluoride) (PVDF) and its copolymers poly(vinylidene fluoride-co-trifluoroethylene) (PVDF-TrFE), poly(vinylidene fluoride-co-hexafluoropropylene) (PVDF-HFP), and poly(vinylidene fluoride-cochlorotrifluoroethylene) (PVDF-CTFE), due to their excellent properties such as high polarity and the possibility of controlling the porosity of the materials through binary and ternary polymer/solvent systems, among others. This review presents the recent advances on battery separators based on PVDF and its copolymers for lithium-ion batteries. It is divided into the following sections: single polymer and co-polymers, surface modification, composites, and polymer blends. Further, a critical comparison between those membranes and other separator membranes is presented, as well as the future trends on this area.

## 1. Introduction

In the field of mobile applications, the efficient storage of energy is one of the most critical issues, since there is a fundamental need to maximize the amount of energy stored. This issue can be accomplished by increasing the gravimetric and volumetric energy density of the batteries [1].

The electrochemical lithium ion battery is used to provide power to a large variety of mobile appliances, such as smartphones, tablets, and laptops, as well as an increasing number of sensors and actuators, which will have a fundamental role in the shaping of the Internet of Things and Industry 4.0 concepts, the main trend for current technological evolution [2]. Lithium ion batteries can also power electric and hybrid vehicles, and take part in the management of renewable energy production, being essential in a more sustainable energy paradigm. As some renewable resources, such as solar and wind, are intermittent over time, storing energy for their use during periods of lack of resources is a critical issue for lithium ion batteries [3,4].

Lithium ion batteries are very suitable for the aforementioned applications due to their advantages with respect to other battery types, as they are lighter, cheaper, have a higher energy density (250 Wh·kg^−1^, 650 Wh·L^−1^), lower charge lost, no memory effect, a prolonged service-life, and a higher number of charge/discharge cycles [5].

Furthermore, the global market of lithium ion batteries is currently growing, and it is expected that in 2022, the market value will reach $46.21 billion, with an annual growth rate of 10.8% [6].

The first commercial lithium ion battery, which was by Sony, entered the market in 1991, with the fundamental contribution of John Goodenough in the development of LiCoO_2_ as the active material for the cathode [7].

**Figure 1:**
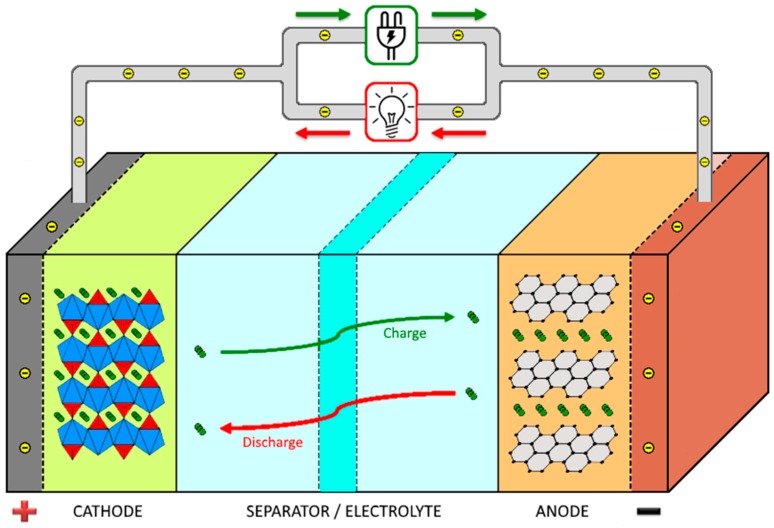
Schematic representation of a lithium ion battery and its working operation.

The main components of a battery are the anode, the cathode, and the separator, which are represented in Figure 1, together with the working principle of a lithium ion battery.

During the discharge process of the battery, the cathode acts as an oxidizing element, receiving electrons from the external electric circuit and being reduced. The anode is the reducing element, releasing electrons to the external electrical circuit, being oxidized during the electrochemical reaction [8].

## 2. Battery Separator: Function, Characteristics, and Types

**Figure 2:**
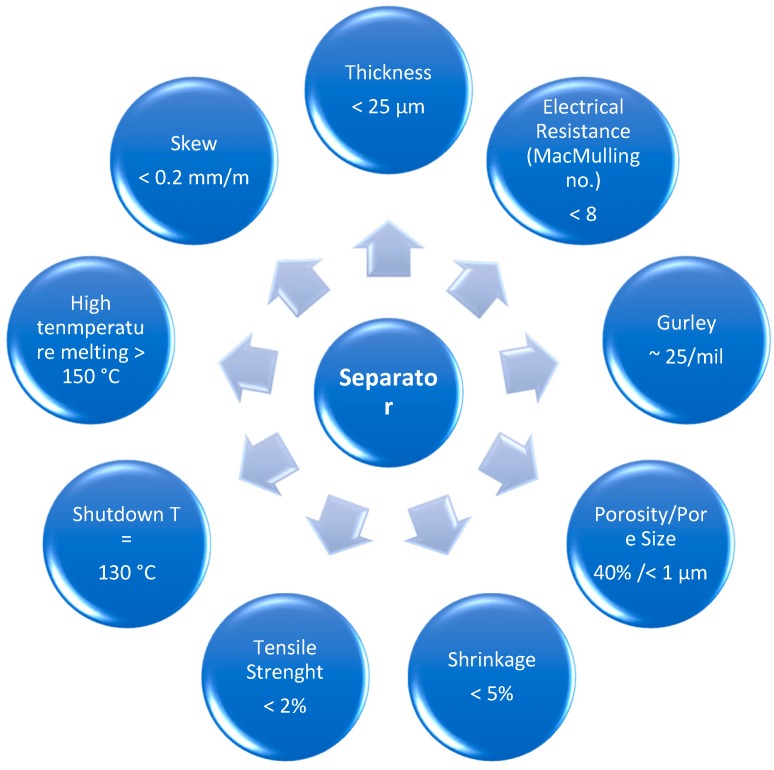
Ideal values for the main requirements of a separator membrane.

Separators play a key role in the operation of electrochemical devices. The main purpose of the separator membranes is to separate the cathode from the anode, avoiding the occurrence of short circuits, and controlling to the mobility of lithium ions between electrodes. The performance of a separator in a lithium ion battery is determined by some requirements such as porosity, chemical and thermal stability, electrical insulator, wettability, dimensional stability, and resistance to degradation by chemical reagents and electrolytes (Figure 2) [9]. Figure 2 shows the ideal values for the main requirements of a separator membrane. 

There are different types of separators, but the most widely used consist of a polymer matrix embedded by the electrolyte solution, i.e., a liquid electrolyte where salts are dissolved in solvents, water, or organic molecules. The main types of separators are shown in Table 1 [10].

The most commonly used materials as matrix for lithium ion battery separators are polymers, or polymer composites. Some of the most commonly used polymers are poly(propylene) (PP), poly(ethylene) (PE), poly(vinylidene fluoride) (PVDF) and its copolymers, poly(ethylene oxide) (PEO), and poly(acrylonitrile) (PAN) [11]. Some separators are developed by blending two different polymers to improve the characteristics of the membrane. In some cases, nanoparticles are added to the matrix as fillers to increase its mechanical stability or ionic conductivity. In composites separators, the most widely used fillers are oxide ceramics (ZrO_2_ [12,13], Al_2_O_3_ [14,15], SiO_2_ [16,17]), carbonaceous fillers (graphene [18], carbon black [19], carbon nanofiber [20]), and ionic liquids [21], among others.

The solvents must possess some requirements to ensure proper battery operation. The properties of a good solvent are high dielectric constant, low viscosity, high chemical stability, and in liquid form over a wide temperature range. For this application, solvents of ethylene carbonate (EC), propylene carbonate (PC), dimethyl carbonate (DMC), diethyl carbonate (DEC), and ethyl methyl carbonate (EMC) are the most commonly used [11].

## 3. Poly(vinylidene fluoride) and Its Copolymers 

Considering the different polymer matrices used for battery separators, PVDF and its copolymers (poly(vinylidene fluoride-co-trifluoroethylene), poly(vinylidene fluoride-co-trifluoroethylene) (PVDF-TrFE), poly(vinylidene fluoride-co-hexafluoropropylene), poly(vinylidene fluoride-co-hexafluoropropylene) (PVDF-HFP), and poly(vinylidene fluoride-cochlorotrifluoroethylene) (PVDF-CTFE)) show exceptional properties and characteristics for the development of battery separators, highlighting high polarity, excellent thermal and mechanical properties, wettability by organic solvents, being chemically inert and stable in the cathodic environment, and possessing tailorable porosity through binary and ternary solvent/non-solvent systems [22,23]. The main properties of these polymers are presented in Table 2 [11].

PVDF and its copolymers are partially fluorinated semi-crystalline polymers where the amorphous phase is located between the crystalline lamellae arranged in spherulites. It can crystallize in different crystalline phase, depending on the temperature and processing conditions [24,25]. In relation to the crystalline phases of PVDF and its copolymers, the most important phases are the β-phase, since it presents ferroelectric, piezoelectric, and pyroelectric properties, and the α-phase, which is the most stable thermodynamically, when material is obtained directly from the melt [24]. As illustrated in Table 2, PVDF and its polymers are characterized by excellent mechanical properties, good thermal stability up to 100 °C, and a high dielectric constant, which is essential for assisting the ionization of lithium salts.

PVDF copolymers have drawn increasing attention for battery separators, as the addition of other monomers to the VDF blocks increases the fluorine content and decreases the degree of crystallinity (Table 2), which is particularly relevant once the uptake of the electrode solution occurs in the amorphous region through a swelling process for accommodating the electrolyte and, as a result, increases the ionic conductivity [29]. The recent literature on PVDF and its battery separator copolymers is structured into four sections dedicated to single polymers, surface modification, composites, and polymer blends, respectively.

The main achievement for PVDF and co-polymers as battery separators was thoroughly reviewed in [11]. Since then, important contributions have been achieved, which are the subject of the present review. 

### 3.1. Single Polymer and Co-Polymers

As already mentioned, one of the main characteristics of PVDF and its co-polymers is their high dielectric permittivity, providing a large affinity with polar electrolytes when compared to other polymers [11]. The main characteristics of the developed PVDF and the copolymer membranes are shown in Table 3.

Table 3 shows that the electrospinning technique is widely used to produce functional membranes. Thus, electrospun separators have been developed for PVDF-PDA [31], PVDF-HFP [44], and PVDF-CTFE [47]. 

**Figure 3:**
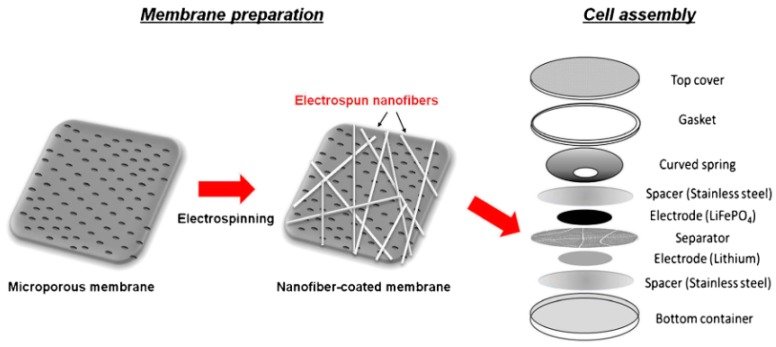
Manufacturing of a testing cell based on PVDF-CTFE separators [47], with copyright permission from Springer Nature.

For the PVDF-CTFE membrane, the cell assembly considered for the battery performance tests is represented in Figure 3.

For PVDF-HFP electrospun membranes, it has been demonstrated that a single layer membrane shows good porosity and uptake value, but that the mechanical stability is negatively affected, with the viscosity of the solution playing an important role [44]. Also, a novel gel electrolyte was developed based on PVDF-HFP by the addition of disiloxane into the electrolyte solution [42], leading to a thermally stable separator that is not flammable, thus contributing to safer lithium ion batteries [45]. It this sense, ionic liquids have also been used in electrolyte solutions, improving both safety and the ionic conductivity of the membranes [43].

A multistep electrospinning technique for the production of PVDF membranes for electrical double-layer capacitors has been proposed, allowing for the manufacture of thinner and more densely packed separators [30].

Further, membranes have been developed based on PVDF for air-cathode in microbial fuel cells [38] and piezo-supercapacitors [39]. Dual asymmetric PVDF separators were produced by a thermally-induced phase separation method, in which the large and interconnected pores in the bulk structure ensures an improved electrolyte uptake and ionic conductivity, while the small pores in the surfaces prevent the loss of electrolyte and the growth of lithium dendrites. It is indicated that those separators ensure safer batteries with high discharge capacity and longer cycle life [36].

A further step towards the development of more environmentally friendly PVDF separator membranes was proposed by using DMPU as a solvent for PVDF, and IL [C2mim][NTf2] as an electrolyte. The use of the IL increased the ionic conductivity and discharge capacity of the membrane when compared with separators using conventional electrolytes [9]. 

**Figure 4:**
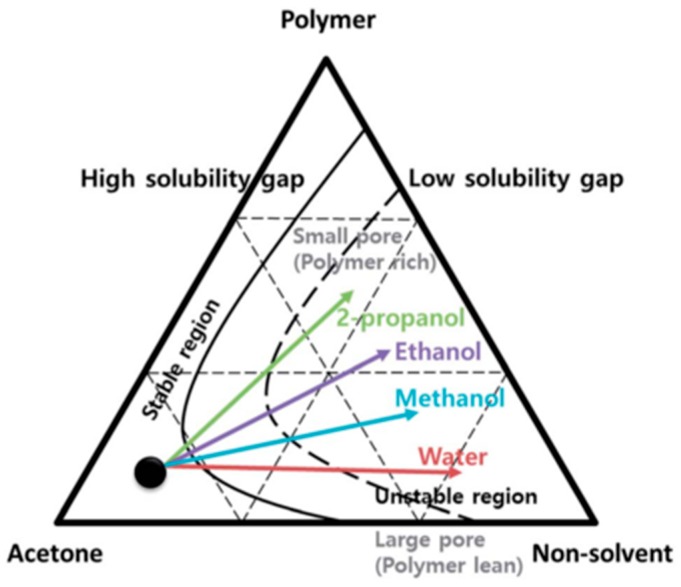
Phase diagram of the ternary mixture—PVDF–HFP, acetone, and non-solvent—in order to control PVDF-HFP membrane morphology [46], with copyright permission from the Royal Society of Chemistry.

Porous PVDF-HFP membranes were prepared with non-solvents using the phase inversion technique. When selecting different types of non-solvents such as water, methanol, ethanol, and propanol, and their contents in acetone, it was possible to control the size of the pores (Figure 4) [46].

Finally, a correlation between the β-phase content of the separators, and the rate capability and cyclability of the batteries was demonstrated for different PVDF co-polymers, showing that the PVDF-TrFE membrane has the best battery performance for the highest β-phase content (100%) [41].

Thus, it is observed that for single (co)polymer membranes, the main focus is to tailor the morphology to obtain good uptake without mechanical deterioration, and to improve the interaction between the electrolyte solution and the separator membrane.

### 3.2. Surface Modification of the Separator Membranes

Typically, surface modification of the membranes is carried out to improve specific properties such as wettability, and thermal and mechanical stability. PVDF membranes have been prepared after different surface modifications, but also have been used to modify the properties of other polymer membranes, as presented in Table 4.

The most commonly used surface modification is the use of PVDF and its copolymers for the coating of other polymers such as polyethylene porous separators. Thus, the coating of PE with a Al_2_O_3_ ceramic layer and a PVDF electrospun nanofiber layer leads to enhanced electrolyte uptake, improved capacity discharge, and cycle life [55]. Similarly, a PDA coating on PVDF improves hydrophilicity, enhancing electrolyte uptake and ionic conductivity of the separator [31].

A typical surface modification technique, such as plasma treatment, allows significant improvement of the electrolyte uptake of PVDF electrospun membranes [48]. 

A hot-pressing technique was proposed to develop PET/PVDF separators, with improved mechanical behavior properties [52].

The preparation of a PVDF/PMMA/PVDF separator showed great potential for its use in lithium-sulfur batteries, showing high initial discharge capacity and cycle stability, also reducing cell polarization and suppressing the shuttle effect, which is described as the transport of soluble polysulfides between both electrodes and the associated charge [54].

A composite membrane with a PVDF/HEC/PVDF sandwich structure was developed, leading to higher electrolyte uptake, ionic conductivity, and cycling performance. It is also greener and safer because of the fire-retardant behavior of its components [58].

For PVDF-HFP membranes, several coatings have been applied, such as ZrO_2_ nanoparticles [64], PP polymer [59], PMMA polymer [60], PDA layer [12], and SiO_2_-modified PET [61], leading mainly to improved electrolyte uptake.

Surface modifications are also achieved by modifying the drying temperature of PVDF-HFP/PET separators prepared by dip-coating, with a drying temperature of 80 °C improving cycle and rate performances with respect to batteries with a conventional PP separator [58]. 

**Figure 5:**
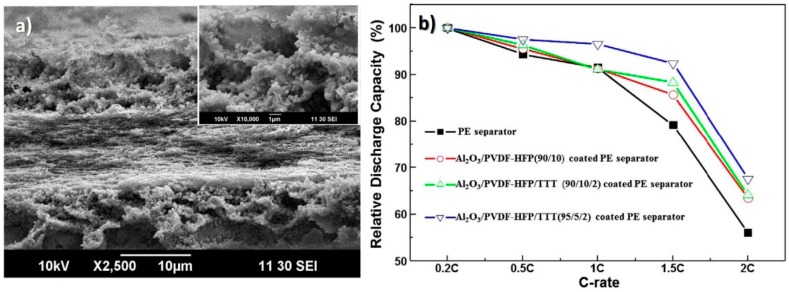
(**a**) Cross-section scanning electron microscopy (SEM) images of the γ-Al_2_O_3_/PVDF-HFP/TTT(95/5/2)- coated PE separator and (**b**) relative discharge capacities as a function of the C-rate [13], with copyright permission from Elsevier.

The dip-coating of a PE separator with γ-Al_2_O_3_/PVDF-HFP/TTT, proved to increase electrolyte uptake and ionic conductivity when compared with conventional membranes, as shown in Figure 5 where its microstructure and cycling performance are presented. The discharge performance was also enhanced as well as the thermal resistance [13].

Basically, surface modifications are essential for improve the electrolyte wettability of the separators, and are realized in several polymer membranes of single and multiple layers with many polymers (PP, PET, PMMA, etc.) and filler nanoparticles.

### 3.3. Composite Membranes

Polymer composites are used to improve battery performance by incorporating suitable fillers, such as oxides ceramic, zeolites, and carbon nanotubes, among others, with the objective of increasing ionic conductivity, mechanical strength, and thermal stability. The main properties of composite separator membranes based on PVDF and its copolymers are presented in Table 5.

Several fillers such as n-butanol [90], SiO_2_ [103], ZnO [86] MgAl_2_O_4_ [105], and MMT [107] particles were used with PVDF and its copolymer composites in order to improve thermal and mechanical stability as well as the ionic conductivity value. 

Mechanical improvement of separators has been achieved by developing sandwich-type composite separators, by a successive electrospinning method and based on PMIA [79].

The addition of DNA-CTMA in a PVDF matrix allows the development of flexible membranes, with interesting mechanical properties, highlighting its favorable stretch properties, allowing foldable separators with elevated elasticity [71].

The addiction of cellulose nanoparticles in the separator structure proved to significantly increase the mechanical strength of the membrane. It also improves the wettability and induces the β-phase formation in PVDF. However, the presence of NCC reduces the ionic conductivity of the membrane [73].

The use of SiO_2_ nanoparticles in a PVDF electrospun separator can raise the mechanical strength of the membrane, thus leading to a more tough and durable battery [106].

Improved security operation for lithium ion batteries, due to suitable flammability resistance, has been addressed by developing PVDF/LiPVAOB composites membranes [33].

The direct application of a ceramic suspension of PVDF/Al_2_O_3_ in the electrode, resulting in a separator-cathode assembly, enhances the adhesion between these structures, and improves electrochemical cell performance [66]. 

PVP/PVDF membranes incorporated with carbon black nanoparticles were produced for supercapacitor applications. The separators showed improvements in mechanical properties and dielectric constant values [19].

GPEs based on boron-containing cross-linker proved to have high thermal resistance, maintaining their dimensional stability up to 150 °C, due to their stable PVDF matrix. Also, ionic conductivity and electrochemical stability were improved when compared to commercial separators [68].

Studies on the influence of solvents in nanoclay/PVDF separators showed that using DMAc as a solvent improves the porosity and electrolyte uptake of the membrane when compared with most used solvents such as NMP or DMF. Furthermore, the addition of PVP to the separator structure contributed to increase the pore size and to reduce the degree of crystallinity [72].

The addition of a metal-organic framework to a polymer structure proved to increase the conductivity of the produced membrane without needing an electrolyte. The membrane also showed high durability and good mechanical properties [76].

The dipping of PVDF nanofiber membranes into Al_2_O_3_ proved to improve the thermal stability of the produced separator and its ionic conductivity. It also shows a low discharge capacity decay, even at high discharge rates [111].

A double-layer separator was prepared with PVDF and reduced graphene oxide, for lithium-sulfur batteries. It is shown that the two layers combined their properties to enhance the thermal stability of the membrane and the cycling performance of the cells [82].

The use of inorganic fibers as substrate for separators lead to improved thermal and mechanical stability when compared to commercial membranes. It was also proven that it enhanced the electrochemical performance of lithium ion cells [115].

CNF/PVDF composite membranes showed greater performance when applied in Li-S batteries, with enhanced cycling stability. The produced batteries retained a capacity of 768.6 mAhg^−1^ after 200 cycles at a 0.5 C rate [20]. The development of PVDF-C separators by the phase-inversion method for Li-S batteries also leads to outstanding electrochemical performance results, associated with the presence of the conductive carbon network in the polymer matrix [69].

**Figure 6:**
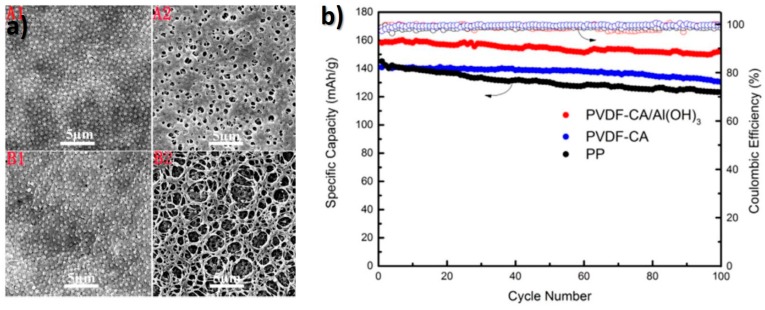
(**a**) SEM images of separators microstructure and (**b**) cycle performance of cells assembled [70], with copyright permission from Elsevier.

In the search for more environmental friendly materials, a separator with PVDF, cellulose acetate and Al(HO)_3_ particles was developed by non-solvent induced phase separation (NIPS), the microstructure being presented in Figure 6a. This membrane exhibited high porosity, electrolyte uptake, and ionic conductivity, as well as good cycling capacity, even at high C-rates, as demonstrated in Figure 6b [70].

PVDF was also used in the study of the potential of zeolitic imidazolate framework-4 in separators. The prepared membranes showed high thermal stability, porosity, ionic conductivity, and cycling performance when compared with conventional separators [120]. 

The incorporation of Meldrum’s acid groups in the PVDF structure proved to increase the ionic conductivity of the membrane, as well as the cycling performance, in particular at high C-rates [74].

PVDF/PFSA electrospun nanofibers allow the development of membrane with high mechanical stability and ionic conductivity with high discharge capacity and cycling stability [81].

A GPE membrane was developed by blending PVDF with PEO and ZrO_2_. This membrane showed high electrolyte uptake, and excellent rate performance and discharge capacity for application in lithium-sulfur batteries [88].

Electrospun membranes with Octaphenyl-POSS nanoparticles showed a significant improvement in porosity and electrolyte uptake. For a ratio of 2:100 (*w*:*w*), the separator proved to have high mechanical stability, ionic conductivity, and thermal stability [77].

A nonaflate anion-based IL and lithium salt was introduced on a GPE, allowing the development of a membrane with high thermal stability and electrochemical properties. When used alongside with a LiCoO_2_ cathode, this separator also showed good discharge capacity and capacity of retention [96].

The addition of MgAl_2_O_4_ as filler in electrospun fibrous PVDF-HFP separators contributes to improving electrochemical performance, with high discharge capacity and excellent cycle life results [104].

The integration of m-SBA15 as filler in a polymer matrix, on the other hand, is advantageous as it decreases the degree of crystallinity of PVDF-HFP, increasing electrolyte uptake and enhancing the ionic conductivity [109,110].

The enhancement of the electrochemical performance has been extensively addressed by composites membranes with TiO_2_ nanoparticles [119], and clay nanosheets [95], the latter improving the interfacial areal connection between the polymer structure and clay, facilitating ion transport.

The NaA zeolite is considered to be a very interesting material for incorporation as filler in lithium ion battery separators. It allows the formation of voids in the composite separator structure, which are filled with electrolyte, substantially increasing the ionic conductivity [108].

The safety operation of lithium ion batteries can be upgraded by the addition of metal hydroxides such as Al(OH)_3_ and Mg(OH)_2_, in PVDF-HFP composite separators. These metal hydroxides endow a fire-retardant behavior to the cells, due to their natural thermal stability [91].

Kuo et al. synthesized an oligomeric ionic liquid from a phenolic epoxy resin. By blending this ionic liquid with PVDF-HFP, a high performance, non-flammable gel polymer membrane was obtained. This membrane exhibits high ionic conductivity, although with a low liquid electrolyte uptake (<50%) [111].

The addiction of ZrO_2_ filler increases the porosity, ionic conductivity, and thermal resistance of the PVDF membranes. The presence of polar constituents and high connected interstitial voids facilitate electrolyte absorption, increasing the ionic conductivity and the performance of the membranes [113]. When a layer of ZrO_2_ was added between two layers of PVDF-HFP, the obtained separator presented even better electrochemical properties [114].

Graphene oxide nanosheets incorporated during the phase inversion of PVDF-HFP improve electrochemical battery performances of the produced separators, as well as thermal stability and the mechanical properties of the membrane [97].

HMSS/PVDF-HFP composite separators with improved porosity were developed; the presence of SiO_2_ spheres created a well-developed microporous structure, leading to higher wettability and ionic conductivity [98].

The incorporation of a superfine LLTO in a PVDF-HFP separator enhanced the ionic conductivity of the membrane. It was also been shown that a cell with a this type of separator presents improved discharge capacity and rate performance [101].

Bohemite composite separators were produced, exhibiting cycling performances comparable to the conventional ones. These membranes are also safer because of the limitation to Li dendrite formation, preventing the occurrence of short circuits [67].

A comparative study of Al_2_O_3_ and NaAlO_2_ particles in a gel polymer electrolyte proved that NaAlO_2_ membranes present higher ionic conductivity than Al_2_O_3_, as well as improved mechanical properties [14].

ZrO_2_ membranes with PVDF-HFP as a binder were produced by solvent casting methods. These separators present high porosity and thermal stability, but show lower mechanical strength than commercially available membranes [116].

A GPE produced by thermal crosslinking of PEGDA and PEGMEA proved to be compatible with lithium ion batteries, with a high coulombic efficiency of 94% after 100 cycles [78].

Liu et al. produced a GPE with PVDF-HFP and graphene via NIPS. The addition of a small concentration of graphene (0.002 wt. %) proved to significantly improve the properties of the membrane by increasing porosity, electrolyte uptake, ionic conductivity, and cycling performance, when compared to commercial separators [18].

Regardless of the fillers type used, Table 5 shows that most of the work is devoted to increasing ionic conductivity and electrochemical performance compared to the pure matrix. In particular, inert oxide ceramics (Al_2_O_3_, TiO_2_, SiO_2_, ZrO_2_) reduce the degree of crystallinity, and enhance mechanical properties and ionic conductivity value. Carbon materials (CNF, Graphene, rGO) improve safety and interfacial stability between electrodes and separator membranes, and lithium fillers such as Li_1,3_Al_0,3_Ti_1,7_(PO_4_)_3_, LiTSFI, and LLTO increase ionic conductivity value of the separators.

In addition, there are other fillers types such as zeolites and clays that are being intensely used for the development of separators, allowing the improvement of electrochemical behavior.

### 3.4. Polymer Blend Separator Membranes

Finally, another type of separator membrane are polymer blends where two different polymers with complementary properties are used; for example one showing excellent mechanical properties and the other with a hydrophilic character. The main properties of polymer blends based on PVDF and its copolymer are presented in Table 6.

PVDF composite separators with methyl cellulose as host of gel polymer electrolyte allows the development of low cost and environmentally friendlier separators with excellent mechanical, thermal, and electrochemical performances [123]. 

A trilayer porous membrane of PVDF-HFP with PVC as the middle layer was developed. It was shown that a good porosity and uptake value can be achieved, though the mechanical stability was negatively affected [44].

Cells produced with PVDF-NCC separators presented good battery performance at high C-rates, which is very critical for meeting the minimum and maximum power-assist requirements for integration in hybrid electric vehicles [124,125].

A mechanically strengthened electrospun composite PVDF-HFP/PEG/PEGDMA separator was developed. PEG and PEGDMA allow the improvement of the mechanical strength of the composite membrane, which is confirmed by the existence of physical bonded structures [143].

P(MMA-co-PEGMA) and PDMS-g-(PPO-PEO) copolymers within PVDF allow the reduction of the crystallinity of the PVDF matrix, and gently improve the electrolyte uptake, thus leading to an enhanced ionic conductivity [129,136].

PLTB can be successfully used in a PVDF-HFP composite separator. In comparison with a typical PP separator, it is more safe and efficient, due to its thermal and electrochemical stability. This separator is very promising in terms of security operation, because of flame retardant characteristics [144].

**Figure 7:**
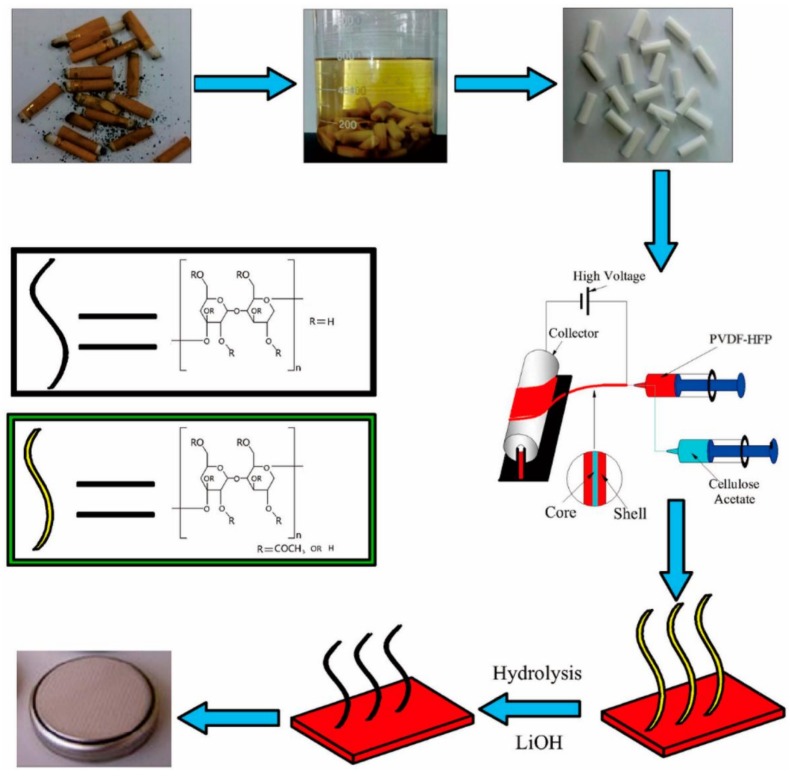
Preparation of PVDF-HFP/CA nanofiber separators for lithium ion batteries [140], with copyright permission from the American Chemical Society.

An eco-friendly technique to recover cellulose acetate from wasted cigarette filters (Figure 7) was developed, and the material can be integrated in a PVDF/CA membrane for lithium ion batteries, which presents a good performance [140].

PVDF separators were manufactured by a phase inversion technique, with two different cross-linking agents (TAIC and MEP) and with the application of gamma radiation. The produced membranes are characterized by good mechanical behavior and low electrical resistance [34]. 

Electrospun PVDF membranes blended with PMMA/SiO_2_ showed good porosity and elevated electrolyte uptake [137]. Blending with PI further enhanced their thermal and mechanical properties, ensuring a better battery performance than commercial PE separators [134].

PVDF/PEO blend membranes show an increase of the ionic conductivity and electrolyte uptake when compared with PVDF membranes. The improved wettability and porosity in x-PEGDA-coated PEI/PVDF membranes has been also reported [147]. 

PVDF-HFP/HDPE membranes were prepared by non-solvent induced phase separation. This separator presents good cycling performance in lithium ion batteries and a high ionic conductivity [141]. Further studies showed an increased discharge capacity of these membranes, by decreasing the size of the HDPE fillers [121].

PVDF/PAN blend separators were produced by TIPS [126] and electrospinning [127], with improved thermal and mechanical properties. The best PVDF/PAN ratio was 90:10. Despite the lower ionic conductivity when compared with conventional separators, these membranes showed higher cycle and C-rate performance [126].

PVDF/PAN electrospun membrane have excellent dimensional stability even at high temperatures, high electrolyte uptake and ionic conductivity, and superior discharge capacity [127].

The blending of PVDF and PEO in an electrospun membrane proved to increase significantly the electrolyte uptake of the separator, while decreasing the shutdown temperature [132]

Cross-linked PBA/PVDF GPE were prepared by soaking semi-interpenetrating polymer networks with liquid electrolyte. For a PBA/PVDF ratio of 1:0.5, the best results of electrolyte uptake, ionic conductivity, and cycling stability were obtained [128].

A PVDF/PET hybrid separator was produced via a mechanical pressing process. The obtained membrane presented high wettability and electrolyte uptake, while maintaining good thermal stability [133].

The introduction of PANI in a PVDF separator by the breath figure method proved to increase the electrolyte uptake and ionic conductivity of the membrane. The best results were obtained for 30% of PANI, with a uniform pore structure and excellent thermal stability [142].

The use of PVDF-HFP/PVSK membranes in lithium-sulfur batteries has been reported. It has been proved that even small amounts of PVSK (5 wt. %) increase the discharge capacity of the cell and reduce the capacity decay [146].

An increase of the use of natural polymers and biopolymers is observed for the preparation of PVDF and copolymer blends, considering the environmental issues. It is demonstrated in Table 6 that they allow to improve mechanical properties and wettability, and consequently the battery performance. In addition, the use of conductive polymers such as PANI in polymer blends has acquired special attention in recent years, considering that the electrical properties are improved without mechanical deterioration. Typically, the most commonly used PVDF and PVDF-HFP blends are developed with PAN and PEO polymers, allowing the improve thermal and mechanical stability, as well as wettability and ionic conductivity value, respectively.

## 4. Conclusions and Future Trends

In this review, the latest advances in PVDF-based battery separators for lithium-ion battery applications are presented.

Considering the excellent properties of PVDF and its copolymers as a separation membrane and the importance of the role of the battery separator in battery applications, this review was divided into four different sections—single polymers, surface modification, polymer composites, and blends, where, for each category, the improvement of the main properties of the separators’ degree of porosity, uptake value, mechanical and thermal properties, ionic conductivity, and cycling performance, as well as safety and environmental impacts,- for the different developed materials was presented.

In the single polymer category, PVDF and PVDF-HFP stand out as the most commonly applied polymers produced by various processing techniques, with TIPS and electrospinning methods being the most commonly used to tailor microstructure (degree of porosity and pore size) to improve battery performance. 

The number of research papers on surface modifications of the membranes has increased in recent years, as the surface of the polymer membrane strongly affects the uptake process. Surface modification is accomplished by coating hydrophilic polymers or plasma treatment to increase the interaction between the polymer membrane and the electrolytic solution.

Generally, the addition of fillers increases battery performance through the improvement of ionic conductivity in polymer composites, but has not yet demonstrated the best filler for PVDF and its copolymer membranes. The most commonly used fillers are inert oxide ceramics, carbon materials, and lithium fillers. The most improved properties are mechanical properties, interfacial stability, between electrodes and separator membranes and ionic conductivity value, respectively.

In relation to the polymer blends, the appearance of new blends based on natural and conductive polymers within PVDF for battery separators has been observed. 

The blends of PVDF and its copolymers widely used are with PAN and PEO polymers, allowing the improvement of mechanical properties and wettability and electric properties, respectively.

The future trends for single polymer separators are to obtain single polymers with a porosity above 50% but a smaller pore size below 500 nm to prevent dendrite growth. Further, it is expected an increase in the use of ionic liquids as electrolytic solution. In relation to surface modifications, the use of poly (ionic liquids) and natural polymers as a surface modification coating of PVDF polymer membranes will be interesting, considering environmental issues.

With respect to polymer composites, future perspectives are related to improving the interaction between polymer matrix and fillers, in order to optimize filler content without decreasing electrical properties or hindering mechanical stability. Also, the use of more than one filler with complementary properties may be the way for improving cycling performance. 

The progress with respect to polymer blends is related to the scalability of the fabrication process, increasing the interaction and compatibilization of the two polymers. 

In summary, PVDF-based battery separators allow the tailoring of all the properties/characteristics required for a new generation of separator membranes for lithium-ion batteries with high power and excellent cycling performance.

## List of Symbols and Abbreviations

(C_2_H_5_)_3_CH_3_NBF4Triethylmethylammonium tetrafluoroborate[C2mim][NTf2]1-ethyl-3-methylimidazolium bis(trifluoromethylsulfonyl)imideAl(OH)_3_Aluminum hydroxideAl_2_O_3_Aluminum oxideAlO(OH)BohemiteANAcetonitrileBCBoron-containing cross-linkerCACellulose acetateCMCcarboxymethyl celluloseCNFCarbon nanofiberDECDiethyl carbonateDEMDiethoxymethaneDMAcDimethyl acetamideDMCDimethyl carbonateDME1,2-dimethoxyethaneDMFDimethyl formamideDMSODimethyl sulfoxideDNA-CTMADeoxyribonucleic acid-cetyltrimethylammoniumDOL1,3-dioxolaneECEthylene carbonateEMCEthyl methyl carbonateEMImNfO-LiNfO1-ethyl-3- methylimidazolium nonafluoro-1-butanesulfonate/lithium nonafluoro-1-butanesulfonateEMITf1-ethyl 3-methyl imidazolium trifluoromethane sulfonateEMITFSI1-ethyl-3-methyl-imidazolium bis(trifluoromethanesulfonyl)imideEPEthyl propionateEt_4_N-BF_4_Tetraethylammonium tetrafluoroborateinGFGlass fiberGOGraphene oxidesGPEGel polymer electrolyteH_2_SO_4_Sulfuric acidHDPEHigh density polyethyleneHECHydroxyethyl celluloseHMSSHollow mesoporous silica spheresHTPB-g-MPEGHydroxyl-terminated polybutadiene-grafted-methoxyl polyethylene glycolKOHPotassium hydroxideLiClO_4_Lithium percholorateLiCoO_2_Lithium cobalt oxideLiFAPLithium Tris(pentafluoroethane)-trifluorophosphateLiNfO/BMImNfOLithium nonafluorobutanesulfonate/1-butyl-3-me-thylimidazolium nonafluorobutanesulfonateLiNO_3_Lithium nitrateLiPF_6_Lithium hexafluorophosphateLiPVAOBLithium polyvinyl alcohol oxalate borateLi-SLithium-sulfurLiTFSIlithium bis(trifluoromethanesulfonyl)imideLLTOLi0.33La0.557TiO3MAMeldrum’s acidMCMethyl celluloseMEPEthylene oxide-propylene oxideMg(OH)_2_Magnesium hydroxideMgAl_2_O_4_Magnesium aluminateMMTMontmorilloniteMOF-808Zirconium (IV) metal-organic frameworkm-SBA 15Mesoporous silicaNaANaA zeoliteNaClO_4_Sodium perchlorateNaTfSodium trifluoromethane sulfonateNCCNanocrystalline celluloseNIPSNon-solvent induced phase separationNMPN-methyl-2-pyrrolidoneOILOligomeric ionic liquid (bromide bis(tri-fluoromethane)sulfonimide)P(MMA-co-PEGMA)Poly(methyl methacrylate-co-poly(ethylene glycol) methacrylate)PANPolyacrylonitrilePANIPolyanilinePBAPoly(butyl acrylate)PCPropylene carbonatePDAPolydopaminePDMS-g-(PPO-PEO)Poly(dimethylsiloxane)-graft-poly(propylene oxide)-block-poly(ethylene oxide)PEPolyethylenePEGPolyethylene glycolPEGDAPoly(ethylene glicol)diacrylatePEGDMAPolyethylene glycol dimethacrylatePEGMEAPoly(ethylene glycol) methyl ether acrylatePEIPolyetherimidePEOPolyethilene oxidePETPolyethylene terephthalatePFSAPerflourosulfonic acidPIPolyimidePLTBPolimeric lithium tartaric acid boratePMIAPoly(m-phenylene isophthalamide)PMMAPolymethyl methacrylatePOSSPolyhedral oligomeric silsesquioxanePPPolypropyleneP-PAEKPhenolphthaleyne-poly(aryl ether ketone)PSx-PEO3Polysiloxane-comb-propyl(triethylene oxide)PSUPoly(sulfone)PTFEPoly(tetrafluoroethylene)PVAPolyvinyl alcoholPVCPoly(vinyl chloride)PVDFPoly(vinylidene fluoride)PVDF-co-CTFEPolyvinylidene fluoride-co-chlorotrifluoroethylenePVDF-co-HFPPoly(vinylidene fluoride-co-hexafluoropropylene)PVDF-HFPPoly(vinylidene fluoride-co-hexafluoropropene)Poly(vinylidene fluoride-hexafluoropropylene)PVDF-PEPolyvinylidene difluoride-coated polyethylenePVDF-TrFEPoly(vinylidene fluoride-trifluoroethylene)PVPPolyvinylpyrrolidonePVSKPolyvinylsulfate potassium saltrGOReduced graphene oxideSCPCSelf-charging power cellSiO_2_Silicon dioxideSNSuccinonitrileSnO_2_Tin oxideTAICTriallyl isocyanurateTEABF_4_Tetraethyl ammonium tetrafluoroborateTiO_2_Titanium dioxideTIPSThermal-induced phase separationTTT1,3,5-trially-1,3,5-triazine-2,4,6(1 H,3 H,5 H)-trioneVCVinylene carbonatex-PEGDAx-polyethylene glycol diacrylateZnOZinc oxideZrO2Zirconium dioxide

## Figures and Tables

**Table 1:** Types and characteristics of different separators adapted from [10].

Separator	Characteristics	Typical Materials
Microporous	Operates at low temperatures (<100 °C); pore size = 50–100 Å	Nonwoven fibers (cotton, nylon, polyester, glass), polymers (PP, PE, PVC, PTFE), rubber, asbestos, wood
Nonwoven	Resistance to degradation by electrolytes; thickness > 25 µm; pore size = 1–100 µm	Polyolefins (PE, PP, PA, PTFE; PVDF; PVC)
Ion exchange membrane	High chemical resistance; impervious to electrolytes; pore size < 20 Å	PE, PP, Teflon-based films
Supported liquid membrane	Solid matrix with a liquid phase; insolubility in electrolyte; high chemical stability	PP, PSU, PTFE, CA
Polymer electrolyte	Simultaneously separator and electrolyte; high chemical and mechanical integrity	Polyethers, PEO, PPO, lithium salts
Solid ion conductor	simultaneously separator and electrolyte	-

**Table 2:** Main properties of PVDF and its copolymers [26,27,28].

Polymer	Melting Temp./°C	Degree of Crystallinity/%	Young Modulus/MPa	Dielectric Constant
PVDF	~170	40–60	1500–3000	6–12
PVDF-TrFE	~120	20–30	1600–2200	18
PVDF-HFP	130–140	15–35	500–1000	11
PVDF-CTFE	~165	15–25	155–200	13

**Table 3:** Separator membranes based on PVDF and co-polymers, indicating also the main properties, and the main goal/achievement of the investigation.

Materials	Electrolyte Solution	Porosity and Uptake (%)	Conductivity (S·cm^−1^) and Capacity (mAh·g^−1^)	Main Goal/Achievement	Reference
PVDF	1 M (C_2_H_5_)_3_CH_3_NBF_4_ + AN	-/-	-/-	Study of multistep electrospinning technique on the fabrication of PVDF composite membranes; High specific power.	[30]
PVDF	1 M LiPF_6_ in EC:DEC (1:1, *w*/*w*)	-/816	6.83 × 10^−4^/101.1 (0.5C)	Performance comparison with a PVDF-PDA separator; Enhanced cycling performance.	[31]
PVDF	1 M LiPF_6_ in EC:DEC (1:1, *v*/*v*)	7/-	-/-	Analysis of the migration mechanism of cation and anions through the separator; The separator allows the control of structural stability and ion mobility.	[32]
PVDF	1 M LiPF_6_ in EC/DMC/EMCC (1:1:1, *w*/*w*/*w*)	-/-	-/95 (0.2C)	Production of a PVDF membrane; Good capacity retention.	[33]
PVDF	1 M TEABF_4_ in AN/PC and 1 M LiPF_6_ in EC/DEC	80/-	1.8 × 10^−2^ (25 °C)/-	Manufacturing of a PVDF separator; Favorable mechanical properties.	[34]
PVDF	1 M LiBF_4_ in EC/DMC (50:50 wt. %)	-/-	4.17 × 10^−3^ (20 °C)/-	Comparison of PVDF membrane performance with Nafigate separators.	[35]
PVDF	1 M LiPF_6_ in EC/DMC/DEC (1:1:1)	78.9/427	1.72 × 10^−3^/164.3 (C/5)	Synthesis of dual asymmetric structure separators; Improved electrolyte uptake and ionic conductivity.	[36]
PVDF	1 M LiPF_6_ in EC/DMC/DEC (1:1:1)	-/-	-/447.36 (0.3C)	Production of a solid state SCPC with a PVDF separator; High storage capacity and stability.	[37]
PVDF	-	-/-	-/-	Assembly of a PVDF separator for air-cathode as application in microbial fuel cells; Improved electricity generation.	[38]
PVDF	PVA/H_2_SO_4_	-/-	-/-	Production of a PVDF separator for piezoelectric supercapacitors; High mechanical strength and elevated capacitance.	[39]
PVDF	1 M NaClO_4_ in EC/DEC (1:1)	81/34	7.38 × 10^−4^ (29 °C)/153	Production of an electroactive electrospun PVDF separator for sodium ion batteries.	[40]
PVDF	1 M LiPF_6_ in EC/DEC (1:1)	70/66	1.5 × 10^−3^/102 (2C)	Study of the effect of different PVDF copolymers as lithium ion battery separators. Demonstration of the relevance of β-phase content.	[41]
PVDF-TrFE	1 M LiPF_6_ in EC/DEC (1:1)	72/84	1.1 × 10^−3^/118 (2C)		
PVDF-HFP	1 M LiPF_6_ in EC/DEC (1:1)	56/79	1.3 × 10^−1^/107 (2C)		
PVDF-CTFE	1 M LiPF_6_ in EC/DEC (1:1)	59/80	1.5 × 10^−3^/85 (2C)		
PVDF	[C_2_mim][NTf_2_]	20/98	2.3 × 10^−4^ (25 °C)/74.6 (C/5)	Preparation of PVDF separators using a green solvent and ionic liquid as the electrolyte.	[9]
PVDF-HFP	LiTFSI	48/248	5.2 × 10^−5^ (20 °C)/-	Application of disiloxane-based electrolytes on PVDF-HFP for the production of gel electrolyte separators; Good thermal and mechanical stability.	[42]
PVDF-HFP	LiNfO/BMImNfO	-/-	2.61 × 10^−2^/(100 °C) 138.1 (C/4)	Production of ionic liquid gel polymer electrolytes; High ionic conductivity.	[43]
PVDF-HFP	1 M LiPF_6_ in EC/DMC (1:2)	70/247	3.2 × 10^−3^ (25 °C)/-	Evaluation of the performance of PVDF-HFP, as a single polymer membrane. Understanding of the method of avoiding the formation of beads in the nanofibers of PVDF-HFP; Good electrolyte uptake.	[44]
PVDF-HFP	1 M LiPF_6_ in EC:DMC (1:1)	78/86.2	1.03 × 10^−3^/145 (0.2C)	Development of a PVDF-HFP gel polymer electrolyte membrane with honeycomb type porous structure; Excellent electrochemical performance.	[45]
PVDF-HFP	1 M LiPF_6_ in EC/DEC/EMC (1:1:1)	-/-	-/-	Production of separators with controlled pore structure; Improved rates and cycling performances.	[46]
PVDF-CTFE	1 M LiPF_6_ in EC:DMC:EMC (1:1:1, *v*:*v*)	74/-	7.51 × 10^−4^ (25 °C)/147 (0.2C)	Preparation of a nanofiber-coated composite separator by electrospinning;High discharge capacity and good cycling stability.	[47]

**Table 4:** Surface modifications on PVDF and co-polymers, indicating also the main properties, goal and achievement.

Materials	Electrolyte Solution	Porosity and Uptake (%)	Conductivity (S·cm^−1^) and Capacity (mAh·g^−1^)	Main Goal/Achievement	Ref
PVDF (plasma-treated)	1 M LiPF_6_ in EC/DMC (1:1)	-/1200	-/-	Study of the effect of plasma treatment in PVDF separators; Improved electrolyte uptake and mechanical properties.	[48]
PE/PVDF	1 M LiPF_6_ in EC:EMC:DEC (1:1:1, *w*:*w*:*w*)	-/-	0.89 × 10^−3^ (25 °C)/-	Investigation into the pore formation process in a coating layer for separators; Enhanced ionic conductivity.	[49]
PE/PVDF	1.10 M LiPF_6_ in EC/PC/EP (3:1:6, *v*:*v*:*v*)	-/-	-/1436 (0.2C)	Study of the electrochemical performance of PE/PVDF separators; Enhanced cycling performance.	[50]
PVDF/PP	1 M LiPF_6_ in EC/DMC (1:1)	58/140	5.9 × 10^−4^/145 (0.5C)	Coating of PVDF particles in the surface of a PP membrane; Increased electrolyte uptake.	[51]
PET/PVDF	1 M LiPF_6_ in EC/DEC/DMC (1:1:1, *w*/*w*/*w*)	-/-	8.36 × 10^−3^/-	Investigation of the performance of a hot-pressed PET/PVDF separator; Excellent mechanical behavior.	[52]
PVDF/HEC	1 M LiPF_6_ in EC/DMC/EMC (1:1:1)	-/135.4	8.8 × 10^−4^ (25 °C)/140	Preparation of a PVDF/HEC/PVDF membrane with a sandwich structure; High electrolyte uptake and ionic conductivity.	[53]
PVDF/PMMA	1 M LiTFSI in DME/DOL (1:1)	-/294	1.95 × 10^−3^ (25 °C)/1711.8	Preparation of a sandwiched GPE based on PVDF and PMMA for lithium-sulfur batteries; High discharge capacity and cycle stability.	[54]
PDA/PVDF	1 M LiPF_6_ in EC:DEC (1:1, wt:wt)	-/1160	9.62 × 10^−4^/104.5 (0.5C)	Prove that the PDA coating can be promising for manufacturing electrospun nanofiber separators; Better cycling performance and elevated power capability.	[31]
PE/(PVDF/Al_2_O_3_)	1 M LiPF_6_ in EC/DEC (1:1)	60.3/125, 314	1.14-1.23 × 10^−3^/-	Development of a multilayer coating for separators; Improvement of thermal stability and electrolyte wetting.	[55]
PI/PVDF/PI	1 M LiPF_6_ in EC/DEC/DMC (1:1:1)	83/476	3.46 × 10^−3^/114.8 (0.5C)	Production of an electrospun sandwich-type separator; Superior porosity, electrolyte uptake, and ionic conductivity.	[56]
PVDF-HFP	1 M NaClO_4_ in EC/PC (1:1)	-/-	3.8 × 10^−3^/291.1 (0.2C)	Development of a PVDF-HFP-coated GF separator for sodium ion batteries; Good cycling performance.	[57]
PVDF-HFP	1 M LiPF_6_ in DMC/EMC/EC (1:1:1)	53.5/106.9	8.34 × 10^−4^/131.33 (5C)	Study of the effect of the drying temperature on the performance of the separator.	[58]
PP/(PVDF-HFP/SiO_2_)	1 M LiPF_6_ in DEC/EC (1:1, *v*/*v*)	-/-	7.2 × 10^−4^/-	Analysis on the effect of a PVDF-HFP/SiO_2_ coating layer for PP separators; Better electrolyte uptake and ionic conductivity.	[59]
PMMA/PVDF-HFP	1 M LiPF_6_ in EC:DMC (1:1)	-/342	1.31 × 10^−3^/143 (0.2C)	Investigation and analysis of a produced PMMA/PVDF-HFP electrolyte membrane; Exceptional thermal and electrochemical stability.	[60]
PVDF-HFP/PDA	LiPF_6_ in EC/DEC/DMC (1:1:1)	72.8/254	1.40 × 10^−3^ (20 °C)/-	Production of a PVDF-HFP/PDA separator by a dip-coating method.	[12]
PVDF-HFP/PET	1 M LiClO_4_ in DMSO	-/282	6.39 × 10^−3^ (25 °C)/158 (0.1C)	Combination of PVDF-HFP with a SiO_2_ nanoparticle-modified PET matrix; Improved thermal stability, electrolyte uptake, and ionic conductivity.	[61]
PP/(AlO_2_/PVDF-HFP)	1 M LiPF_6_ in EC/DEC (1:1*, v*/*v*)	-/-	7.95 × 10^−4^/98.6 (0.2C)	Inspection of the performance of a separator for PP membrane coating; Improved thermal stability.	[62]
γ-Al_2_O_3_/PVDF-HFP/TTT	1 M LiClO_4_ in EC/DEC (1:1)	-/157	1.3 × 10^−3^/~100 (0.5C)	Dip coating of a PE separator with γ-Al_2_O_3_/PVDF-HFP/TTT; Increased electrolyte uptake and ionic conductivity.	[13]
PP/PE/PP/PVDF-co-CTFE	1 M LiPF_6_ in EC/DMC/DEC (1:1:1, *v*:*v*:*v*)	-/-	-/-	Fabrication of PVDF-co-CTFE nanofiber coatings for improving the performance of polyolefin separators; High electrolyte uptake and good wettability.	[63]

**Table 5:** Polymer composites based on PVDF and co-polymers with main properties, goal, and achievement.

Materials	Fillers	Electrolyte Solution	Porosity and Uptake (%)	Conductivity (S·cm^−1^) and Capacity (mAh·g^−1^)	Main Goal/Achievement	Ref.
PVDF	Al_2_O_3_	1 M LiPF_6_ in EC/DEC/DMC (1:1:1)	55.8/153.5	2.23 × 10^−3^ (25 °C)/114.2	Production of a composite PVDF/Al_2_O_3_; High thermal stability and ionic conductivity, low discharge capacity decay.	[15]
PVDF	Al_2_O_3_	EC/DMC (1:1)	-/230	1.24 × 10^−3^/151.97 (C)	Core-shell composite nonwoven separator of PVDF-HFP@Al_2_O_3_; high heat resistance up to 200 °C without any shrinkage,	[65]
PVDF	Al_2_O_3_	1 M LiPF_6_ in EC/DEC (1:1, *v*:*v*)	67/230	1.49 × 10^−3^/146.3 (0.2C)	Separator-cathode assembly with PVDF/Al_2_O_3_; Good electrochemical performance.	[66]
PVDF	AlO(OH) nanoparticles	1 M LiPF_6_ in EC/DEC (3:7)	-/65	-/-	Ceramic separator based on boehmite nanoparticles; Improved safety and wettability.	[67]
PVDF	BC	1 M LiTFSI in EC/DEC (1:1)	-/-	4.2×10^−3^ (30 °C)/-	Preparation of GPEs based on cross-linkers; High ionic conductivity and thermal stability.	[68]
PVDF	Carbon	1 M LiTFSI and 0.1 M LiNO_3_ in DOL/DME (1:1)	-/-	-/827 (0.5C)	PVDF-C separator by phase inversion technique; Superior rate performance and stability.	[69]
PVDF	CNF	1 M LiTFSI in DOL/DME (1:1)	-/119	-/1739.2 (C)	Production of CNF/PVDF separators for Li-S batteries Great battery discharge capacity and cycling stability.	[20]
PVDF	Cellulose acetate/Al(OH)_3_	1 M LiPF_6_ in EC/DMC/EMC (1:1:1)	68.6/403.9	2.85 × 10^−3^/151.97 (C)	Environmental friendly materials in a separator; High electrolyte uptake, ionic conductivity and cycling performance.	[70]
PVDF	DNA-CTMA	LiAsF_6_ in EC/EMC/DMC	-/-	-/-	PVDF/DNA-CTMA membrane as a solid polymer/gel electrolyte separator; Improved thermal and mechanical properties.	[71]
PVDF	LiPVAOB	1 M LiPF_6_ in EC/DMC/EMCC (1:1:1, *w*:*w*:*w*)	-/88.5	2.6 × 10^−4^/120 (0.2C)	Composite gel polymer electrolyte PVDF/LiPVAOB membrane; Good ionic conductivity.	[33]
PVDF	Nanoclays/PVP	1 M LiPF_6_ in EC/DMC (1:1)	87.4/553.3	-/-	Study of the influence of solvents in the separator High porosity and uptake.	[72]
PVDF	NCC	1 M LiPF_6_ in EC/DMC (1:1)	-/-	3.73 × 10^−3^ (25 °C)/-	Preparation of NCC-PVDF separators by phase inversion; Improved wettability and mechanical properties.	[73]
PVDF	MA groups	1 M LiPF_6_ in EC/DMC/EMC (1:1:1)	67.4/-	1.48 × 10^−3^/136 (0.2C)	Study of the addition of MA groups to the PVDF structure; High ionic conductivity.	[74]
PVDF	MMT	1 M LiPF_6_ in EC/EMC/DEC (1:1:1)	84.08/333	4.20 × 10^−3^ (25 °C)/144	Effect of different contents of MMT filler in PVDF separators; High ionic conductivity and porosity.	[75]
PVDF	MOF-808	-	-/-	1.56 × 10^−4^ (65 °C)/-	Production of a MOF/polymer membrane; Good mechanical properties and durability.	[76]
PVDF	Octaphenyl-POSS	1 M LiPF_6_ in EC/DMC/EMC (1:1:1)	66.1/912	4.2 × 10^−3^/145.8 (0.5C)	Electrospun membrane with octaphenyl-POSS particles; Increased uptake and porosity, high ionic conductivity.	[77]
PVDF	Polyether (PEGDA+PEGMEA)	1 M LiPF_6_ in EC/DMC/EMC (1:1:1)	-/230	~1.4 × 10^−3^ (25 °C)/93 (0.5C)	Preparation of GPEs with PVDF and polyethers.	[78]
PVDF	PMIA	1 M LiPF_6_ in EC/DMC/EMC (1:1:1, *w*:*w*:*w*)	-/-	8.1 × 10^−4^/135.29 (0.2C)	Composite sandwich type separator, by electrospinning; High capacity retention and good rate performance.	[79]
PVDF	P-PAEK	1 M LiPF_6_ in EC/DMC (1:1)	71.7/123.7	/141.6 (C/2)	Development of a P-PAEK/PVDF separator High wettability and electrolyte uptake.	[80]
PVDF	PFSA	1 M LiPF_6_ in EC/DMC/EMC (1:1:1)	-/-	1.53 × 10^−3^/137.9 (C)	PVDF/PFSA blend membrane; High stability and discharge capacity.	[81]
PVDF	rGO	1 M LiTFSI + 0.1 M LiNO_3_ in DME/DOL (1:1)	71/380	/646	Double-layer PVDF/rGO membrane by electrospinning; High safety and cycling stability.	[82]
PVDF	SiO_2_	1 M LiPF_6_ in EC/DMC/EMC (1:1:1)	54.1/279.5	-/175.7	Synthesis of a composite separator with SiO_2_; High wettability, uptake and thermal/mechanical stability.	[17]
PVDF	SiO_2_	1 M LiPF_6_ in EC/EMC (1:1 in volume)	70/370	2.6 × 10^−3^/132 (C)	Addition of SiO_2_ nanoparticles on PVDF membranes; Improvement of wettability and ionic conductivity.	[83]
PVDF	SiO_2_	1 M LiPF_6_ in EC/DEC (1:1, *v*:*v*)	85/646	7.47 × 10^−3^/159 (0.2C)	Electrospun PVDF/SiO_2_ composite separator; Excellent thermal stability and high ionic conductivity.	[84]
PVDF	SnO_2_	1 M LiPF_6_ in EC/DMC (1:1 *w*:*w*)	-/-	-/-	Use of SnO_2_ nanoparticles in a PVDF electrospun separator; Good cycling performance.	[85]
PVDF	ZnO	1 M LiPF_6_ in EC/EMC (1:2)	-/-	-/-	Piezo-separator for integration on a self-charging power cell; Enhanced electrochemical performance.	[86]
PVDF	ZnO	1 M LiPF_6_ in EC/DEC (1:1)	-/-	-/-	Piezo-separator for self-charging power cells; Stable and efficient performance.	[87]
PVDF	ZrO_2_/PEO	1 M LiTFSI in DOL/DME (1:1)	-/147.3	3.2 × 10^−4^ (25 °C)/1429 (0.2C)	GPE for lithium-sulfur batteries; High discharge capacity and rate performance.	[88]
PVDF-HFP	Al_2_O_3_	0.5 M NaTf/EMITf	-/-	6.3–6.8 × 10^−3^ (25 °C)/-	Introduction of Al_2_O_3_ in a gel polymer electrolyte; Improved mechanical properties.	[14]
PVDF-HFP	Al_2_O_3_	1 M LiPF_6_ in EC/DEC +2% VC	-/372	1.3 × 10^−3^/155 (0.5C)	Colloidal Al_2_O_3_ composite separator; enhancement of the mechanical strength of the PVDF-HFP separator.	[89]
PVDF-HFP	Al_2_O_3_	1 M LiPF_6_ in EC/DMC/EMC (*v*:*v*:*v* = 1:1:1)	-/420	4.7 × 10^−4^/109 (4C)	Production of a low cost membrane, with a simple and easy scalable manufacturing process; High electrolyte uptake and good electrochemical stability and performance.	[90]
PVDF-HFP	Al(OH)_3_	1.15 M LiPF_6_ in EC/EMC (3:7, *v*:*v*)	84/127	10^−3^/81 (C/2)	Upgrading the battery safety operation by the addition of metal hydroxides in composite separators; Suitable electrolyte uptake.	[91]
PVDF-HFP	Al_2_O_3_/CMC	1 M LiPF_6_ in EC/DEC/PC/EMC (2:3:1:3)	42.7/-	9.3 × 10^−4^ (25 °C)/-	Composite separator with Al_2_O_3_/CMC; Safer and more stable separators.	[92]
PVDF-HFP	BN	1 M LiPF_6_ in EC/DEC (1:1)	-/-	-/150 (0.2C)	3D separator; improved cycling stability with lower voltage polarization	[93]
PVDF-HFP	CA	1 M LiPF_6_ in EC/DMC	85/310	1.89 × 10^−3^/136 (8C)	Porous and honeycomb-structured membrane; higher lithium-ion transference number and improved rate performance	[94]
PVDF-HFP	Clay	1 M LiPF_6_ in EC/DEC/EMC (1:1:1, *v*:*v*:*v*)	-/-	1.49 × 10^−3^/-	New technique to incorporate clay sheets in a PVDF-HFP matrix, as separator; Thermal stability and higher ionic conductivity.	[95]
PVDF-HFP	EMImNfO-LiNfO	-	-/-	3.92 × 10^−4^/(20 °C) 57 (C)	Introduction of anion-based IL and lithium salt in a GPE; High thermal stability, good electrochemical properties.	[96]
PVDF-HFP	GO	1 M LiPF_6_ in EC/DEC/EMC (1:1:1)	-/71	1.115 × 10^−3^ (25 °C)/-	Addition of GO in separators to increase thermal properties; improved electrochemical and mechanical properties.	[97]
PVDF-HFP	Graphene	1 M LiPF_6_ in EC/DMC/EMC (1:1:1)	88/470	3.61 × 10^−3^/149 (C)	PVDF-HFP/graphene GPE by NIPS; Increased porosity, uptake and ionic conductivity.	[18]
PVDF-HFP	HMSS	1 M LiPF_6_ in EC/DEC (1:1)	~70/285	2.57 × 10^−3^ (25 °C)/-	Development of PVDF-HFP with HMSS separators; Increased wettability and porosity.	[98]
PVDF-HFP	Li_1,3_Al_0,3_Ti_1,7_(PO_4_)_3_	1 M LiTFSI + 0.25 M LiNO_3_ in DME/DOL (1:1)	34/143.9	8.8 × 10^−4^ (25 °C)/1614	Ceramic/polymer membrane for lithium-sulfur cells; High ionic conductivity and discharge capacity.	[99]
PVDF-HFP	LiTSFI/SN	-	-/-	1.97 × 10^−3^ (20 °C)/-	Production of supercapacitors with GO electrodes and GPE; High ionic conductivity.	[100]
PVDF-HFP	LLTO	1 M LiPF_6_ in EC/DMC/EMC (1:1:1)	69.8/497	13.897 × 10^−3^ (25 °C)/155.56	Incorporation of LLTO in a PVDF-HFP separator; Improved ionic conductivity.	[101]
PVDF-HFP	PI	1 M LiPF_6_ in EC/DMC (1:1)	73/350	1.46 × 10^−3^/-	Evaluation of a bicomponent electrospinning method to produce the separator, Good physical properties and improved electrochemical stability.	[102]
PVDF-HFP	PET/SiO_2_	1 M LiPF_6_ in EC/DEC (1:1)	60/-	9.3 × 10^−4^/-	Separator with an organized porous structure, with benefits for cell operation at high C-rates; Excellent cell performance.	[103]
PVDF-HFP	MgAl_2_O_4_	1 M LiPF_6_ in EC:DEC (1:1, *v*:*v*)	-/-	2.80 × 10^−3^/140 (0.1C)	Influence of different quantities of the MgAl_2_O_4_ filler in the membrane; Good ionic conductivity.	[104]
PVDF-HFP	MgAl_2_O_4_	1 M LiPF_6_ in EC/DEC (1:1, *w*:*w*)	60/81	10^−3^ (30 °C)/140 (C/10)	MgAl_2_O_4_ as filler of thin and flexible separator; Good thermal stability and stable cycling performance.	[105]
PVDF-HFP	Mg(OH)_2_	1.15 M LiPF_6_ in EC/EMC (3:7, *v*:*v*)	64/115	8.08 × 10^−4^/105 (C/2)	Upgrading the battery safety operation by the addition of metal hydroxides in composite separators; High thermal stability and good capacity retention.	[106]
PVDF-HFP	MMT	1 M LiPF_6_ in EC/DEC (1:1, *v*:*v*)	40/251	9.01 × 10^−4^/105 (0.1C)	Use of montmorillonite as filler; High thermal stability and stable cycling performance.	[107]
PVDF-HFP	NaA	1 M LiPF_6_ in EC/DEC (1:1, *v*:*v*)	65/194	2.1 × 10^−3^/-	Separator with incorporation of NaA zeolite; Excellent thermal stability and wettability.	[108]
PVDF-HFP	NaAlO_2_	0.5 M NaTf/EMITf	-/-	5.5–6.5 × 10^−3^ (25 °C)/-	Introduction of NaAlO_2_ in a gel polymer electrolyte; Improved ionic conductivity.	[14]
PVDF-HFP	m-SBA15	1 M LiPF_6_ in EC/DEC (1:1)	-/82.83	3.23 × 10^−3^/156 (0.1C)	A PVDF-HFP composite membrane with m-SBA15 as filler; High coulomb efficiency.	[109]
PVDF-HFP	m-SBA15	1 M LiPF_6_ in EC/DEC (1:1)	-/85.36	3.78 × 10^−3^/198.6 (0.1C)	Effect of the addition of a silica filler on a PVDF-HFP composite matrix separator; High coulomb efficiency.	[110]
PVDF-HFP	OIL	1 M LiPF_6_ in EC/DEC (1:1)	-/13	2 × 10^−3^ (25 °C)/141 (C)	Synthesis of OIL from a phenolic epoxy resin; Non-flammability, good cell performance.	[111]
PVDF-HFP	SiO_2_	1 M LiPF_6_ in EC/DMC (1:2)	65.41/217	-/124.5 (C)	Synthesis of dual asymmetric structure separators with SiO_2_ particles; High thermal stability and electrolyte uptake.	[16]
PVDF-HFP	SiO_2_	1 M LiPF_6_ in DMC/EMC/DC/VC (46.08:22.91:27.22:3.79)	26.7/202	8.47 × 10^−4^ (25 °C)/154.4	Composite separator with SiO_2_; Improved thermal stability and cycling performance.	[112]
PVDF-HFP	TiO_2_	1 M LiPF_6_ in EC/DMC/EMC (1:1:1, *v*:*v*:*v*)	58/330	3.45 × 10^−3^/122 (10C)	Evaluation of the performance of a nanocomposite polymer membrane with addition of TiO_2_; Excellent electrochemical performance.	[85]
PVDF-HFP	ZrO_2_	1 M LiPF_6_ in EC/DEC (1:1)	71/182	1.48 × 10^−3^ S·cm^−1^ (25 °C)/126.8 mAhg^−1^ (0.5C)	Preparation of ZrO_2_/PVDF-HFP by the dip-coating method High wettability, ionic conductivity, and thermal resistance.	[113]
PVDF-HFP	ZrO_2_	1 M LiPF_6_ in EC/EMC (1:3)	-/-	2.06 × 10^−3^ (25 °C)/149.7	Improvement of the electrochemical properties of a electrospun membrane High uptake and ionic conductivity.	[114]
PVDF-HFP	ZrO_2_	1 M LiPF_6_ in EC/DEC/DMC (1:1:1)	87.53/351.2	3.2 × 10^−4^/646 (0.2C)	Inorganic fibers as substrates to separators; High thermal stability and good mechanical properties.	[115]
PVDF-HFP	ZrO_2_	1 M LiPF_6_ in EC/DMC (1:1)	60/160	10^−3^ (25 °C)/75 (C)	Development of thin and flexible ZrO_2_ separators High porosity and thermal stability.	[116]
PVDF-HFP	ZrO_2_	1 M LiPF_6_ in EC/DMC (1:1)	95.7/481	2.695 × 10^−3^ (25 °C)/-	Incorporation of ZrO_2_ in PVDF-HFP electrospun membranes; High ionic conductivity and cycling stability.	[117]
PVP/PVDF	Black carbon nanoparticles	6 M KOH	-/-	-/-	Production of separators for supercapacitor applications Improved thermal and mechanical properties.	[19]
PP/PVDF-HFP	PMMA	1 M LiPF_6_ in EC/DMC (1:1, *v*:*v*)	77.9/212	1.57 × 10^−3^/138 (0.2C)	Physical and electrochemical performances of a PP/PVDF-HFP/PMMA composite separator; Enhanced thermal stability and electrolyte uptake.	[52]
PP/PVDF-HFP	SiO_2_	1 M LiPF_6_ in EC/DEC (1:1, *v*:*v*)	-/290	1.76 × 10^−3^/150 (0.2C)	PP/PVDF-HFP separator, with the inclusion of SiO_2_ nanoparticles; Favorable chemical stability and discharge capacity.	[118]
PI/PVDF-HFP	TiO_2_	1 M LiPF_6_ in EC/DEC (1:1, *v*:*v*)	-/-	1.88 × 10^−3^/161 (0.5C)	Electrospun PI/PVDF-HFP membrane, with addition of TiO_2_ nanoparticles; Excellent electrochemical properties.	[119]

**Table 6:** Polymer blends based on PVDF and co-polymers with main properties, goal, and achievement.

Materials	Blends	Electrolyte Solution	Porosity and Uptake (%)	Conductivity (S·cm^−1^) and Capacity (mAh·g^−1^)	Main Goal/Achievement	Ref
PVDF	HDPE	1 M LiPF_6_ in EC/DEC/DMC (1:1:1)	58/260	2.54 × 10^−3^ S·cm^−^^1^ (25 °C)/156.1 mAhg^−^^1^ (0.1C)	Production of a sponge-like PVDF/HDPE film; High ionic conductivity and cycling performance.	[121]
PVDF	HTPB-g-MPEG	1 M LiPF_6_ in EC/DMC/EMC (1:1:1)	56/350	3.1 × 10^−3^/116 (C)	Enhancement of the stability of entrapped liquid electrolyte and corresponding ion conductivity.	[122]
PVDF	MC	1 M LiPF_6_ in EC/DEM/EMC (1:1:1, *w*:*w*:*w*)	-/138.6	1.5 × 10^−3^/110 (C)	PVDF composite separator with cellulose material; Excellent electrochemical performance.	[123]
PVDF	MEP	1 M TEABF_4_ in AN/PC and 1 M LiPF_6_ in EC/DEC	77/-	1.3 × 10^−2^/-	Manufacturing by phase inversion, with MEP as a cross-linking agent; Good mechanical strength.	[34]
PVDF	NCC	1 M LiFAP in EC/DMC (1:1)	-/-	-/-	Separators with applications in hybrid electric vehicles; Favorable performance at high-voltage cells.	[124]
PVDF	NCC	1 M LiPF_6_ in EC/DMC (1:1)	-/-	-/108 (1C)	Separators with applications in hybrid electric vehicles; Influence on high-rate cell operation.	[125]
PVDF	PAN	1 M LiPF_6_ in EC/DMC/DEC (1:1:1)	77.7/414.5	2.9 × 10^−3^ (25 °C)/-	Improved thermal and mechanical properties; High cycling stability.	[126]
PVDF	PAN	1 M LiPF_6_ in EC/DMC/EMC (1:1:1)	-/320	1.45 × 10^−3^/145.71 (0.2C)	Production of an electrospun blended membrane; High thermal and mechanical stability.	[127]
PVDF	PBA	1 M LiPF_6_ in EC/DEC/DMC (1:1:1)	-/120	8.1 × 10^−4^ (25 °C)/95 (0.1C)	Preparation of cross-linked PBA/PVDF GPE; Good cycling stability.	[128]
PVDF	PDMS-g-(PPO-PEO)	1 M LiPF_6_ in EC/DMC/EMC (1:1:1, *w*:*w*:*w*)	80.1/512	4.5 × 10^−3^/120 (1C)	Porous separator; Good electrochemical stability.	[129]
PVDF	PEGDA	1 M LiPF_6_ in EC/DMC (1:1)	-/-	3.3 × 10^−3^/117 (0.1C)	Separator produced by thermal polymerization; High capacity retention.	[130]
PVDF	PEO	1 M LiPF_6_ in EC/DMC (1:1)	/530	-/-	Production of blended membranes by electrospinning; improved conductivity and uptake.	[131]
PVDF	PEO	1 M LiPF_6_ in EC/DMC (1:1)	-/527	-/-	Development of electrospun membranes; High electrolyte uptake, low shutdown temperature.	[132]
PVDF	PET	-	80/270	-/-	Synthesis of a hybrid separator; High wettability and electrolyte uptake.	[133]
PVDF	PI	1 M LiPF_6_ in EC/PC/DEC/VC (35.4:17.2:45.1:2.3)	-/-	1.3 × 10^−3^/141	Preparation of the separator by electrospinning; Improved thermal stability and mechanical properties.	[134]
PVDF	PMMA/CA	1 M LiPF_6_ in EC/DMC (1:1, *w*:*w*)	99.1/323	-/-	Elevated porosity and electrolyte uptake.	[135]
PVDF	P(MMA-co-PEGMA)	1 M LiPF_6_ in EC/EMC/DMC (1:1:1, *w*:*w*:*w*)	-/372	3.01 × 10^−3^/-	Porous separator; Improved capacity retention.	[136]
PVDF	PMMA/SiO_2_	-	80.1/293.2	1.97 × 10^−3^/-	Evaluation of the effect of a PMMA and SiO_2_ blend on a PVDF electrospun membrane as a separator; High electrolyte uptake and improved ionic conductivity.	[137]
PVDF	PVP	1 M Et_4_N-BF_4/_PC	-/360	1.8 × 10^−3^ (25 °C)/-	Separators for supercapacitors; High uptake and power density.	[138]
PVDF	TAIC	1 M TEABF_4_ in AN/PC and 1 M LiPF_6_ in EC/DEC	75/-	1.4 × 10^−2^/-	Manufacturing of separator by phase inversion, with TAIC as cross-linking agent. High ionic conductivity.	[34]
PVDF-TrFE	PEO	1 M LiTFSI in PC	44.5/107	5.4 × 10^−4^/124 (C/5)	Research on the physical and chemical properties of a PVDF-TrFE/PEO blend Favorable cycling performance.	[139]
PVDF-HFP	CA	1 M LiPF_6_ in EC/DMC/EMC (1:1:1, *v*:*v*:*v*)	66.36/355	6.16 × 10^−3^/138 (0.2C)	Investigation of the use of CA from waste cigarette filters, in PVDF-HFP membranes; Good electrochemical performance and excellent thermal stability.	[140]
PVDF-HFP	HDPE	-	71/300	2.97 × 10^−3^ (25 °C)/140.5 (C)	Preparation of the separator by non-solvent-induced phase separation; High ionic conductivity.	[141]
PVDF-HFP	PANI	1 M LiPF_6_ in EC/DMC (1:1)	83/270	1.96 × 10^−3^/-	High thermal stability, electrolyte uptake, and ionic conductivity	[142]
PVDF-HFP	PEG/PEGDMA	1 M LiClO_4_ in EC/DEC (1:1, *v*:*v*)	71/212	1.70 × 10^−3^/-	Investigation into a strengthened electrospun nanofiber membrane separator; High porosity and electrolyte uptake.	[143]
PVDF-HFP	PLTB	1 M LiPF_6_ in EC/DMC (1:1, *v*:*v*)	70/260	1.78 × 10^−3^/138 (0.5C)	Excellent electrochemical performance.	[144]
PVDF-HFP	PSx-PEO3	1 M LiTFSI in EC/DMC (1:1, *w*:*w*)	-/520	4.2 × 10^−4^ (20 °C)/123 (C)	Production of a safe PVDF-HFP blended membrane, which can be sprayed; Elevated electrolyte uptake.	[145]
PVDF-HFP	PVSK	1 M LiTFSI + 0.25 M LiNO_3_ in DME/DOL (1:1)	27/-	-/1220	Improved cycling performance.	[146]
PVDF-HFP	PVC	1 M LiPF_6_ in EC/DMC (1:2)	62/230	1.58 × 10^−3^/125 (0.1 C)	Tri-layer polymer membrane; Good mechanical and thermal stability.	[44]
PEI/PVDF	x-PEGDA	1 M LiPF_6_ in EC/DMC/EMC (1:1:1)	64.6/235.6	1.38 × 10^−3^ (25 °C)/160.3 (0.2C)	Production of x-PEGDA-coated PEI/PVDF membranes; high wettability, porosity, and ionic conductivity.	[147]
